# Clinically Diagnosed Insomnia and Risk of All-Cause and Diagnosis-Specific Disability Pension: A Nationwide Cohort Study

**DOI:** 10.1155/2013/209832

**Published:** 2013-12-31

**Authors:** Catarina Jansson, Kristina Alexanderson, Göran Kecklund, Torbjörn Åkerstedt

**Affiliations:** ^1^Division of Insurance Medicine, Department of Clinical Neuroscience, Karolinska Institutet, Berzelius väg 3, 6th Floor, 171 77 Stockholm, Sweden; ^2^Stress Research Institute, Stockholm University, 106 91 Stockholm, Sweden

## Abstract

*Background*. Insomnia and disability pension are major health problems, but few population-based studies have examined the association between insomnia and risk of disability pension. *Methods*. We conducted a prospective nationwide cohort study based on Swedish population-based registers including all 5,028,922 individuals living in Sweden on December 31, 2004/2005, aged 17–64 years, and not on disability or old age pension. Those having at least one admission/specialist visit with a diagnosis of disorders of initiating and maintaining sleep (insomnias) (ICD-10: G47.0) during 2000/2001–2005 were compared to those with no such inpatient/outpatient care. All-cause and diagnosis-specific incident disability pension were followed from 2006 to 2010. Incidence rate ratios (IRRs) and 95% confidence intervals (CIs) were estimated by Cox regression. *Results*. In models adjusted for prior sickness absence, sociodemographic factors, and inpatient/specialized outpatient care, associations between insomnia and increased risks of all-cause disability pension (IRR 1.35, 95% CI 1.09–1.67) and disability pension due to mental diagnoses (IRR 1.86, 95% CI 1.38–2.50) were observed. After further adjustment for insomnia medications these associations disappeared. No associations between insomnia and risk of disability pension due to cancer, circulatory, or musculoskeletal diagnoses were observed. *Conclusion*. Insomnia seems to be positively associated with all-cause disability pension and disability pension due to mental diagnoses.

## 1. Introduction

Insomnia is defined as complaint of or difficulty initiating or maintaining sleep or experiencing nonrestorative sleep that impairs daily social, occupational, or other functioning [1, 2]. Insomnia is a large and increasing health problem worldwide [3–5], associated with substantial costs for individuals, employers, and society [3]. The prevalence of insomnia in the adult population ranges from 4 to 50%, while fewer, that is, about 6–15%, are diagnosed with insomnia [1, 2, 4, 6]. The etiology of sleep disorders such as insomnia is multifactorial [7] and studies have shown that such disorders are associated with older age, female sex, low socioeconomic status (SES), and work-related stress [2, 3, 7, 8]. Moreover, insomnia has been suggested to adversely influence quality of life [1], work capacity [9], and endocrinology, immunology, and metabolism [10]. Thus, insomnia is associated with a wide range of health problems and diseases such as hypertension, inflammation, obesity, cardiovascular disease, cognitive and intellectual impairment, and mental disorders, predominantly depression and anxiety [1, 4, 11].

Disability pension is another major health problem, entailing severe social, economic, and health-related consequences for individuals and a considerable economic burden for society [12–14]. About 8% of the Swedish population aged 16–64 years were on disability pension in March 2010 of which disability pensions due to musculoskeletal and mental diagnoses were the most common [14]. Known risk factors for all-cause disability pension include high age, female sex, low SES, being unmarried, living in smaller places, adverse psychosocial and physical working conditions, poor self-rated health, chronic disease, obesity, smoking, and factors measured in late adolescence [14–17]. Moreover, disability pension is generally preceded by sickness absence [12], but the majority of those who are sickness absent are not granted disability pensions.

Despite adverse economic and health-related consequences of both insomnia and disability pension, few studies have focused on the influence of insomnia on early exit from the labor market [6, 9]. However, an association between insomnia and disability pension might be expected [9], potentially mediated by reduced work capacity, poor self-rated health [18], or other diseases [4]. Some prior cohort studies have examined sleep problems/insomnia and risk of disability pension [1, 6, 19, 20]. Existing evidence is, however, inconclusive [6] due to different definitions of outcomes, data sources, social security settings, and response rates [21]. Moreover, most previous studies are based on smaller and/or selected, that is, not population-based, samples, including only self-reported, not clinically diagnosed, sleep problems or insomnia [1, 6, 19, 20, 22, 23]. Thus, the aim of the present study was to—for the first time—examine insomnia diagnosed in inpatient and specialized outpatient care and risk of incident disability pension using a nationwide population-based prospective cohort study including data regarding several potential confounders.

## 2. Methods

### 2.1. Study Design

This prospective cohort study consists of all 5,620,619 individuals who were registered as living in Sweden on December 31, 2004, and December 31, 2005, respectively, and on December 31, 2005, were 17–64 years old. By using the Personal Identity Number (a unique ten-digit number assigned to all Swedish residents) data from the following nationwide, population-based registers were linked: (1) *Longitudinal Integration Database for Health Insurance and Labor Market Studies* (LISA), held by Statistics Sweden, including data for cohort definition, potential confounders (i.e., sociodemographic factors), old age pension, and follow-up regarding migration; (2) *Micro Data for Analysis of the Social Insurance database* (MiDAS), held by the Swedish Social Insurance Agency, including outcome data regarding all-cause and diagnosis-specific disability pension and data regarding old age pension; (3) the *National Patient Register* (PAR); (4) the *Swedish Prescribed Drug Register*; and (5) the *Causes of Death Register*, all held by the National Board of Health and Welfare, including exposure data (i.e., insomnia), potential confounders (i.e., inpatient/specialized outpatient care and medications), and mortality data, respectively. The study was approved by the Regional Ethical Review Board in Stockholm, Sweden.

### 2.2. Insomnia

Exposure data was based on inpatient and specialized outpatient care (PAR data) where inpatient/outpatient diagnoses are classified according to *The International Statistical Classification of Diseases and Related Health Problems, Tenth Revision* (ICD-10). Insomnia was defined as having at least one admission/hospitalization or at least one specialist visit with a diagnosis of disorders of initiating and maintaining sleep (insomnias) (ICD-10, chapter VI: G47.0). Inpatient care was based on admissions during 2000–2005 and specialized outpatient care on visits during 2001–2005 (i.e., nationwide outpatient care data, not including primary care, available since 2001). We constructed five different exposure variables regarding insomnia in- and outpatient care with main and/or secondary diagnoses, studied separately and combined, dichotomized a priori into (i) no insomnia (G47.0) in-/outpatient care during 2000–2005/2001–2005 (unexposed/reference group) and (ii) at least one admission/specialist visit with insomnia (G47.0) during 2000–2005/2001–2005.

### 2.3. Incident Disability Pension

The Swedish social insurance system includes sickness benefits covering up to 80% of lost income when work capacity is reduced due to disease or injury among all adult residents with income from work or unemployment benefits. Disability pension may be granted when a disease or injury has led to permanent work incapacity, covering up to 64% of lost income. Old age pension is mostly granted at 65 years but may be granted earlier. To identify all incident disability pensions we used MiDAS, comprising all disability pensions granted among Swedish residents since 1993. Disability pensions were defined as incident disability pensions received during follow-up, 2006–2010. Disability pension diagnoses are classified according to ICD-10. We analyzed all-cause disability pension and diagnosis-specific disability pension due to common diagnostic groups regarding disability pension and/or common chronic diseases. Thus, the following main diagnoses were studied: (i) malignant and benign tumors (ICD-10, chapter II: C00-C97, D00-D48), (ii) mental and behavioral disorders (ICD-10, chapter V: F00-F99), (iii) diseases of the circulatory system (ICD-10, chapter IX: I00-I99), and (iv) diseases of the musculoskeletal system and connective tissue (ICD-10, chapter XIII: M00-M99).

### 2.4. Exclusions of Cohort Members

The cohort included 5,620,619 individuals. After excluding 30 individuals who were erroneously registered as alive in 2005, 28,131 individuals with early old age pension starting before or at January 1, 2005, and 563,536 individuals with ongoing or newly granted disability pension in 2005, respectively, 5,028,922 individuals were included in the statistical analyses.

### 2.5. Statistical Analyses

The cohort members were followed from January 1, 2006 to December 31, 2010, December 31 of the year the participant turned 65, date of emigration, date of death, or date of an incident disability pension, whichever came first. Incidence rate ratios (IRRs) and 95% confidence intervals (CIs) were estimated by Cox proportional hazards models [24], using time since entry into the cohort as underlying time scale and the PHREG procedure in SAS, release 9.2 (SAS Institute Inc. Cary, NC). Data were analyzed in crude and multivariable models. The following potential confounders, that is, known risk factors for insomnia and all-cause disability pension, respectively, were successively adjusted for: *prior sickness absence*, that is, sickness benefits (in three predefined categories: (i) no sickness benefits, (ii) 1–179 sick-leave days, and (iii) 180+ sick-leave days); *age* (in 10-year intervals, reference group “17–24 years”); *sex* (reference group “men”); *education *(in three categories, reference group “high educational level that is more than 12 years”); *region of residence* (in three categories, reference group “larger cities”); *summarized number of hospitalization days*, that is, inpatient data, and *summarized number of specialist visits*, that is, outpatient data (i.e., two variables in three categories: (i) 0 hospitalization days/visits (reference group), (ii) ≤median hospitalization days/visits, and (iv) >median hospitalization days/visits); and finally *medical treatment for diseases of the nervous system, that is, psycholeptics/insomnia medications*, that is, prescribed, dispensed, and purchased drugs classified according to the following Anatomical Therapeutic Chemical (ATC) codes: antipsychotics (N05A), anxiolytics (N05B), and hypnotics and sedatives (N05C) (dichotomized as (i) no purchased antipsychotics, anxiolytics, or hypnotics and sedatives and (ii) at least one purchase of prescribed antipsychotics, anxiolytics, or hypnotics and sedatives). Prior sickness absence was based on sickness benefits during 2003–2005, sociodemographic factors on registration on December 31, 2005, inpatient care on admissions during 2000–2005, specialized outpatient care on visits during 2001–2005, and insomnia medications on purchases during July–December 2005 (i.e., nationwide data available since July 2005). Summarized number of hospitalization days/visits was based on main ICD-10 diagnoses only and in- and outpatient diagnoses regarding normal delivery, singleton (ICD-10, 080); chapter XVI (perinatal conditions) and chapter XXI (factors of significance for health and for contacts with health care, except for e-codes) were not included. Stratified analyses by sex (as female sex is a risk factor for both insomnia and disability pension) and insomnia medications were also performed. Observations with missing data on any of the covariates included in the models were excluded from the analyses. The number of missing data was, however, few (Tables 1 and 2).

## 3. Results

### 3.1. Characteristics of Study Participants

The 5,028,922 study participants together contributed with almost 24 million person-years at risk of incident disability pension during follow-up, 2006–2010 (Table 2). Insomnia inpatient/specialized outpatient care, that is, the exposure, was rare, including in total only 632 exposed individuals (0.01%). The distributions across the adjusted factors for insomnia inpatient/outpatient care combined, including both secondary + main diagnoses (ICD-10 G47.0), are shown in Table 1. The all-cause disability pension incident rate (IR) was high within all categories of the factors adjusted for and highest in the age category 55–64 years (IR 13.96), among women (IR 7.22), among persons with low educational level (IR 9.32), among those living in smaller cities/rural areas (IR 7.05), among those with long-term prior sickness absence (IR 82.30), among those with hospitalization days (IR 19.20) and specialists visits (IR 13.86) above the median, respectively, and among those having at least one purchase of antipsychotics, anxiolytics, hypnotics, or sedatives (IR 36.10) (Table 1). The most common incident disability pension diagnoses during follow-up were mental and musculoskeletal diagnoses (data not shown).

### 3.2. All-Cause Disability Pension

In total, we observed 142,192 incident disability pensions during follow-up, 2006–2010, more among women (82,311, 58%) compared to men (59,881, 42%) (Table 1). Three- to sixfold increased risks of all-cause disability pension were observed among persons having insomnia inpatient or outpatient specialized care regarding all five exposure variables in the crude models (IRR 5.08, 95% CI 4.11–6.28 (insomnia in-/outpatient care combined, main/secondary G47.0 diagnosis)) (Table 2). There were no major differences regarding having insomnia as a main or secondary diagnosis. After successive adjustment for prior sickness absence, sociodemographic factors, and summarized inpatient/specialized outpatient care, the positive associations regarding insomnia inpatient care, secondary diagnosis and insomnia outpatient care, main diagnosis, were no longer significant, while insomnia inpatient care, main diagnosis, insomnia outpatient care, secondary diagnosis, and combined insomnia in-/outpatient care remained, although attenuated (IRR 1.35, 95% CI 1.09–1.67 (insomnia in-/outpatient care, main/secondary G47.0 diagnosis)). After further adjustment for insomnia medications, the positive associations regarding these exposures also became nonsignificant (Table 2). We also adjusted for hypnotics and sedatives separately (i.e., ATC code N05C; hypnotics and sedatives combined), but the influence on the associations was similar as when adjusting for antipsychotics, anxiolytics, hypnotics, and sedatives combined (data not shown). Both hypnotics and hypnotics and sedatives combined were strongly associated with increased risks of all-cause disability pension in the fully adjusted model (hypnotics: IRR 1.97, 95% CI 1.94–2.00, hypnotics and sedatives combined: IRR 2.19, 95% CI 2.16–2.22).

In the stratified analyses, a similarly increased risk of all-cause disability pension was observed among men having insomnia in-/outpatient care in the model adjusted for prior sickness absence, sociodemographic factors, and inpatient/specialized outpatient care (IRR 1.56, 95% CI 1.14–2.13), but this association became nonsignificant after additional adjustment for insomnia medications. Among women, a positive association between insomnia and risk of all-cause disability pension was observed in the model adjusted for prior sickness absence and sociodemographic factors (IRR 1.37, 95% CI 1.02–1.82) that became nonsignificant after further adjustment for in-/outpatient care and insomnia medications. Among the majority having no purchase of prescribed insomnia medications in 2005, there was a positive association between insomnia and risk of all-cause disability pension (IRR 1.51, 95% CI 1.08–2.13), while among the 225,076 individuals having at least one purchase of insomnia medication, no association between insomnia and risk of all-cause disability pension was observed (Table 3).

### 3.3. Disability Pension due to Mental Diagnoses

In total, 55,811 incident disability pensions due to mental diagnoses were observed during follow-up, although only 43 among the exposed. A positive association between insomnia and risk of disability pension due to mental diagnoses was observed after adjustment for prior sickness absence, sociodemographic factors, and in-/outpatient care (IRR 1.86, 95% CI 1.38–2.50 (insomnia in-/outpatient care combined, main/secondary G47.0 diagnosis)), but after further adjustment for insomnia medications this association became nonsignificant (data not shown).

### 3.4. Disability Pension due to Musculoskeletal Diagnoses

In total, 39,941 incident disability pensions due to musculoskeletal diagnoses were observed, although only 12 among the exposed. A positive association between insomnia and risk of disability pension due to musculoskeletal diagnoses was observed in the crude model (IRR 6.14, 95% 4.05–9.31 (insomnia in-/outpatient care combined, main/secondary G47.0 diagnosis)), but after adjustment no association was observed (data not shown).

### 3.5. Disability Pension due to Cancer and Circulatory Diagnoses

In total, 4,630 disability pensions due to cancer diagnoses and 9,876 disability pensions due to circulatory diagnoses were observed. No associations between insomnia and risk of disability pension due to these diagnoses were observed (data not shown).

## 4. Discussion

To our knowledge, this is the first nationwide cohort study of insomnia and risk of disability pension. We observed associations between insomnia and increased risks of all-cause disability pension and disability pension due to mental diagnoses even after adjustment for prior sickness absence, sociodemographic factors, and summarized inpatient/specialized outpatient care in the total cohort and among men analyzed separately. The strongest association was observed regarding disability pension due to mental diagnoses. After adjustment for insomnia medications these associations became nonsignificant. No associations between insomnia and risk of disability pension due to cancer, circulatory, or musculoskeletal diagnoses were observed.

Hitherto, to our knowledge, there are only four previous cohort studies, performed in Norway and Finland and based on smaller or not population-based samples, of insomnia and risk of disability pension [1, 6, 19, 20, 22, 23]. In these studies, positive associations of similar strengths as in the present study between self-reported insomnia/sleep problems and risk of all-cause disability pension and disability pension due to mental diagnoses were observed, although none of these studies adjusted for prior sickness absence or insomnia medications. In contrast to our findings, positive associations between sleep problems and disability pension due to nervous, circulatory, and musculoskeletal diagnoses and injuries were observed in some prior studies [6, 19, 23], which might be due to clinically diagnosed insomnia being rare in our study or our more comprehensive adjustment for potential confounders. That the positive associations became nonsignificant after adjustment for insomnia medications may be due to insomnia being secondary to, for example, depression or other disorders. Another potential explanation is limited power as there were few individuals with a clinical diagnosis of insomnia and/or that those with self-reported insomnia symptoms may have been included in the reference group. Although a low prevalence of insomnia inpatient/specialized outpatient care was expected, the observed figures are extremely low and may suggest presence of underdiagnosis. Thus, for insomnia outpatient care, secondary diagnosis, the point estimate was increased also after adjustment for insomnia medications. In addition, the adjustment for insomnia medications might partly adjust for those treated for insomnia or a mental disorder in primary care as well as inpatient/specialized outpatient care. Hypnotic drugs are among the most widely used treatments in adult medicine, although the indication may not be sleep-related since physicians often use another diagnosis if they believe that insomnia is secondary to other conditions [25] and a cross-sectional association between self-reported sleep disorders and register-based hypnotics (ATC code N05C) has been observed [26]. In the present study, insomnia medications were strongly associated with an increased risk of disability pension. Thus, potential overadjustment due to collinearity between clinically diagnosed insomnia and insomnia medications may have been introduced in our study by the adjustment for hypnotics. In addition, the present study is the first to adjust for prior sickness absence.

In contrast, even after adjustment for inpatient and specialized outpatient care (including, e.g., ICD-10, chapter V; mental and behavioral disorders), we observed a strong positive association between insomnia and risk of disability pension due to mental diagnoses. One potential explanation is that mental disorders might be in the causal pathway/mediators between insomnia and disability pension or that insomnia is an early symptom of a mental disorder which may be the underlying cause of both insomnia and disability pension [10, 26].

Among men, we found a positive association between insomnia and risk of all-cause disability pension after adjustment for prior sickness absence, sociodemographic factors, and in-/outpatient care, potentially explained by some risk factors for disability pension being more common among men with insomnia. That no association between insomnia and risk of disability pension was found among those with at least one purchase of insomnia medications might be due to limited power as the point estimates were increased or that disability pension was more common in the reference group.

An important strength of this study is the population-based nationwide prospective study design, including the whole Swedish population aged 17–64 years, entailing high statistical power and avoiding selection bias. The availability of objectively measured register data regarding clinically diagnosed insomnia, incident disability pension, and covariates with no or very few missing and the possibility to adjust for several potential confounders are other major strengths. The need to adjust for physical and mental disorders when studying consequences of poor sleep has been stressed [20]. The follow-up and detection of incident disability pensions are complete due to the high quality and nationwide coverage of the Swedish population-based registers used [27–29]. Limitations include potential underestimation of the exposure as insomnia inpatient and specialized outpatient care is rare in Sweden because insomnia symptoms often are untreated or treated in primary care. Studies have shown that mild and even severe insomniacs do not always seek help for treatment [2, 3]. This may have resulted in limited statistical power to ascertain weak associations or attenuated the associations observed. Moreover, many individuals with self-reported insomnia symptoms were probably included in our reference, that is, “unexposed,” group, although our exposure definition may have identified patients with the most severe insomnia as insomnia diagnoses are adequately and thoughtfully made by the treating physician. Another potential limitation is that data regarding potential confounders such as adverse life style factors and work-related stress were not included in the nationwide registers, although some of these factors are associated with low SES and should partly be adjusted for by our adjustment for SES based on education.

Early exit from work is a serious challenge for workplaces, employees, and social security and it has been stressed that sleep problems warrant attention in occupational health to prevent reduced work capacity, disability pension, and morbidity [6, 30]. If insomnia symptoms are detected early it may help prevent early exit from work and provide tools for supporting employees continuing their work careers until normal retirement age [6].

## 5. Conclusions

This population-based nationwide cohort study demonstrates increased risks of all-cause disability pension and disability pension due to mental diagnoses among individuals with clinically diagnosed insomnia after adjustment for prior sickness absence, several sociodemographic factors, and inpatient/specialized outpatient care, although these associations disappeared after adjustment for insomnia medications. Thus, early detection of insomnia symptoms may prevent disability pension.

## Figures and Tables

**Table 1:** Number of participants, person-years, all incident disability pensions (DPs), and incidence rates (IRs) by the adjusted factors.

	No. of exposed (%) (insomnia)^b^	No. of participants (%)^a^	Person-years	No. of incident DPs	IR^c^
Age groups (year); Dec. 31, 2005					
17–24	53 (>0)	846,146 (17)	4,133,741	15,026	3.64
25–34	97 (>0)	1,100,539 (22)	5,359,645	12,757	2.38
35–44	138 (>0)	1,182,671 (23)	5,760,797	26,222	4.55
45–54	196 (>0)	1,012,125 (20)	4,889,723	37,901	7.75
55–64	148 (>0)	887,441 (18)	3,601,907	50,286	13.96
Sex; Dec. 31, 2005					
Male	321 (>0)	2,611,409 (52)	12,352,585	59,881	4.85
Female	311 (>0)	2,417,513 (48)	11,393,229	82,311	7.22
Education; Dec. 31, 2005^d^					
High educational level (more than 12 years)	226 (>0)	1,681,493 (33)	4,236,610	29,918	3.73
Medium educational level (10–12 years)	295 (>0)	2,379,667 (47)	11,313,525	68,007	6.01
Low educational level (0–9 years)	110 (>0)	919,722 (18)	8,010,680	39,464	9.32
Missing		48,040 (1)			
Region of residence; Dec. 31, 2005^e^					
Larger cities	257 (>0)	1,896,500 (38)	8,968,370	44,142	4.92
Medium sized cities	215 (>0)	1,806,371 (36)	8,537,511	54,047	6.33
Smaller cities/rural areas	160 (>0)	1,326,051 (26)	6,239,934	44,003	7.05
Prior sickness absence; 2003–2005					
No sickness benefits	319 (>0)	3,949,089 (79)	18,891,108	39,962	2.12
1–179 reimbursed sick-leave days	137 (>0)	833,572 (17)	3,943,005	27,193	6.90
180+ reimbursed sick-leave days	176 (>0)	246,261 (5)	911,700	75,037	82.30
Inpatient care; admission: 2000–2005					
0 hospitalization days	239 (>0)	3,897,245 (78)	18,549,913	75,199	4.05
≤median summarized hospitalization days	160 (>0)	613,853 (12)	2,886,232	22,649	7.85
>median summarized hospitalization days	233 (>0)	517,824 (10)	2,309,668	44,344	19.20
Outpatient care; 2001–2005					
0 specialist visits	15 (>0)	1,823,960 (36)	8,718,627	18,216	2.09
≤median summarized specialist visits	186 (>0)	1,836,577 (37)	8,759,807	37,182	4.24
>median summarized specialist visits	431 (>0)	1,368,385 (27)	6,267,380	86,794	13.86
Antipsychotics, anxiolytics, hypnotics, and sedatives combined^f^; July–Dec. 2005					
No prescribed medications	380 (>0)	4,801,705 (95)	22,792,763	107,788	4.73
At least one purchase of prescribed medications	252 (>0)	227,217 (5)	953,051	34,404	36.10

Total (missing excluded)	567	5,028,922 *(4,980,882) *			

^a^Observations with missing data on any characteristic included in the study were excluded from the estimation of person-years, number of incident DPs and IRs.

^b^Insomnia in-/outpatient care (ICD-10: G47.0, main/secondary diagnosis).

^c^IRs per 100,000 person-years for all-cause DP; follow-up, 2006–2010.

^d^Statistics Sweden derives the attained “highest education” based on information regarding education according to the Swedish Standard Classification of Education. We classified SES based on education into three commonly used categories.

^e^“Region of residence” is based on “H-regions,” that is, homogenous regions regarding the population base, a categorization by Statistics Sweden based on municipalities according to the local and regional population bases following the scale urban-rural. We categorized these regions into three categories.

^f^The Swedish Prescribed Drug Register contains data on drugs (ATC codes) but lacks information on indication of treatment, which prohibits identification of specific disease groups and it is not possible to link drugs bought over-the-counter to individual persons.

**Table 2:** Total cohort. Insomnia and risk of all-cause incident disability pension (DP), Swedish nationwide cohort study; follow-up, 2006–2010^a^.

In-/outpatient care with diagnoses of disorders of initiating and maintaining sleep (ICD-10: G 47.0)	No. of participants (%)	Person-years	No. of DPs	Crude IRR (95% CI)	Adjusted IRR (95% CI)^b^	Adjusted IRR (95% CI)^c^	Adjusted IRR (95% CI)^d^	Adjusted IRR (95% CI)^e^
Insomnia inpatient care (G47.0 main diagnosis)								
0 admissions/hospitalization due to insomnia, 2000–2005 (unexposed)	4,980,748 (100.00)	23,560,231	137,370	1.00 (reference)	1.00 (reference)	1.00 (reference)	1.00 (reference)	1.00 (reference)
At least one admission due to insomnia, 2000–2005	134 (0.00)	584	19	5.29 (3.38–8.30)	1.34 (0.86–2.10)	1.30 (0.83–2.04)	1.20 (1.18–1.21)	0.79 (0.50–1.23)
Insomnia inpatient care (G47.0 secondary diagnosis)								
0 admissions/hospitalization due to insomnia, 2000–2005 (unexposed)	4,980,799 (100.00)	23,560,431	137,382	1.00 (reference)	1.00 (reference)	1.00 (reference)	1.00 (reference)	1.00 (reference)
At least one admission due to insomnia, 2000–2005	83 (0.00)	384	7	3.10 (1.49–6.45)	0.94 (0.45–1.97)	1.18 (0.56–2.47)	0.98 (0.47–2.05)	0.70 (0.33–1.47)
Insomnia outpatient care (G47.0 main diagnosis)								
0 specialist visits due to insomnia, 2001–2005 (unexposed)	4,980,552 (99.99)	23,559,370	137,348	1.00 (reference)	1.00 (reference)	1.00 (reference)	1.00 (reference)	1.00 (reference)
At least one specialist visit due to insomnia, 2001–2005	330 (0.01)	1,445	41	4.70 (3.47–6.37)	1.30 (0.96–1.76)	1.33 (0.98–1.80)	1.17 (0.86–1.58)	0.83 (0.61–1.12)
Insomnia outpatient care (G47.0 secondary diagnosis)								
0 specialist visits due to insomnia, 2001–2005 (unexposed)	4,980,743 (100.00)	23,560,223	137,367	1.00 (reference)	1.00 (reference)	1.00 (reference)	1.00 (reference)	1.00 (reference)
At least one specialist visit due to insomnia, 2001–2005	139 (0.00)	591	22	6.14 (4.05–9.31)	1.89 (1.24–2.87)	2.22 (1.46–3.36)	1.97 (1.30–2.99)	1.50 (0.99–2.27)
Insomnia in-/outpatient care (G47.0 main/secondary diagnosis)								
0 admissions/visits due to insomnia, 2000/2001–2005 (unexposed)	4,980,251 (99.99)	23,558,071	137,304	1.00 (reference)	1.00 (reference)	1.00 (reference)	1.00 (reference)	1.00 (reference)
At least one admission/visit due to insomnia, 2000/2001–2005	631 (0.01)	2,744	85	5.08 (4.11–6.28)	1.47 (1.19–1.82)	1.52 (1.23–1.88)	1.35 (1.09–1.67)	0.96 (0.78–1.19)

Total	4,980,882	23,560,815	137,389					

^a^The numbers of participants, person-years, and incident DPs are from the adjusted models where missing observations were excluded. In the crude models the number of missing observations = 0. The small number of missing observations for education, family situation, and country of birth is shown in Table 1.

^b^IRRs adjusted for prior sickness absence.

^c^IRRs adjusted for prior sickness absence, age, sex, education, and region of residence.

^d^IRRs adjusted for prior sickness absence, age, sex, education, region of residence, inpatient care, and specialized outpatient care.

^e^IRRs adjusted for prior sickness absence, age, sex, education, region of residence, inpatient care, specialized outpatient care, and medications (i.e., antipsychotics, anxiolytics, hypnotics, and sedatives).

**Table 3:** Stratified analyses. Insomnia and risk of all-cause incident disability pension (DP), Swedish nationwide cohort study; follow-up, 2006–2010.

In-/outpatient care with diagnoses of disorders of initiating and maintaining sleep (ICD-10: G 47.0)	No. of participants (%)	Person-years	No. of DPs	Crude IRR (95% CI)	Adjusted IRR (95% CI)^a^	Adjusted IRR (95% CI)^b^	Adjusted IRR (95% CI)^c^	Adjusted IRR (95% CI)^d^
*Subcohort: men *								
Insomnia in-/outpatient care (G47.0 main/secondary diagnosis)								
0 admissions/visits due to insomnia, 2000/2001–2005 (unexposed)	2,585,422 (99.99)	12,254,479	57,137	1.00 (reference)	1.00 (reference)	1.00 (reference)	1.00 (reference)	1.00 (reference)
At least one admission/visit due to insomnia, 2000/2001–2005	320 (0.01)	1,428	39	5.59 (4.09–7.64)	1.52 (1.11–2.08)	1.77 (1.29–2.42)	1.56 (1.14–2.13)	1.08 (0.79–1.47)
*Subcohort: women *								
Insomnia in-/outpatient care (G47.0 main/secondary diagnosis)								
0 admissions/visits due to insomnia, 2000/2001–2005 (unexposed)	2,394,829 (99.99)	11,303,591	80,167	1.00 (reference)	1.00 (reference)	1.00 (reference)	1.00 (reference)	1.00 (reference)
At least one admission/visit due to insomnia, 2000/2001–2005	311 (0.01)	1,316	46	4.76 (3.57–6.35)	1.44 (1.08–1.92)	1.37 (1.02–1.82)	1.21 (0.91–1.62)	0.89 (0.67–1.19)
*Subcohort: no insomnia medications*								
Insomnia in-/outpatient care (G47.0 main/secondary diagnosis)								
0 admissions/visits due to insomnia, 2000/2001–2005 (unexposed)	4,755,427 (99.99)	22,613,214	103,607	1.00 (reference)	1.00 (reference)	1.00 (reference)	1.00 (reference)	
At least one admission/visit due to insomnia, 2000/2001–2005	379 (0.01)	1,723	33	4.10 (2.92–5.74)	1.61 (1.15–2.27)	1.65 (1.17–2.32)	1.51 (1.08–2.13)	
*Subcohort: insomnia medications* ^ e^								
Insomnia in-/outpatient care (G47.0 main/secondary diagnosis)								
0 admissions/visits due to insomnia, 2000/2001–2005 (unexposed)	224,824 (99.99)	944,857	33,697	1.00 (reference)	1.00 (reference)	1.00 (reference)	1.00 (reference)	
At least one admission/visit due to insomnia, 2000/2001–2005	252 (0.01)	1,022	52	1.40 (1.07–1.84)	0.88 (0.67–1.16)	0.91 (0.70–1.20)	0.84 (0.64–1.10)	

^a^IRRs adjusted for prior sickness absence.

^b^IRRs adjusted for prior sickness absence, age, sex (i.e., only in subcohorts for insomnia medications), education, and region of residence.

^c^IRRs adjusted for prior sickness absence, age, sex (i.e., only in subcohorts for insomnia medications), education, region of residence, inpatient care, and specialized outpatient care.

^d^IRRs adjusted for prior sickness absence, age, sex, education, region of residence, inpatient care, specialized outpatient care, and medications (i.e., antipsychotics, anxiolytics, hypnotics, and sedatives).

^e^At least one purchase of antipsychotics, anxiolytics, hypnotics, and sedatives; July–December 2005.
